# Enhancing Tailings Stability with Polymers and Industrial By-Products: An Experimental Study

**DOI:** 10.3390/polym18101196

**Published:** 2026-05-13

**Authors:** Yazeed A. Alsharedah, Aly Ahmed, Fayyaz Ullah, Yasser Altowaijri

**Affiliations:** 1 Department of Civil Engineering, College of Engineering, Qassim University, Buraydah 51452, Saudi Arabia; y.altwaijry@qu.edu.sa; 2 Department of Civil and Environmental Engineering, Western University, London, ON N6A 3K7, Canada; aahme243@uwo.ca; 3 Water Science Institute, Cranfield University, Bedfordshire MK43 0AL, UK

**Keywords:** tailing stabilization, polymer additives, CKD, gypsum, UCS, dam safety, consolidation, shear strength, sustainability

## Abstract

The stability of upstream tailings remains a critical geotechnical challenge due to the inherently weak mechanical properties of fine-grained mine tailings. This study investigated a tailing improvement method using (i) emulsified polymer and (ii) combinations of recycled gypsum and cement kiln dust (CKD). A comprehensive experimental program—including unconfined compressive strength (UCS) analysis, direct shear tests (DSTs), and oedometer consolidation tests—was conducted to assess the performance of various treatment mixtures. The results showed that blends of CKD and gypsum, particularly at a 1:2 ratio and a 10% dosage, significantly improved shear strength, reduced compressibility, and lowered hydraulic conductivity by over an order of magnitude. The inclusion of plaster (commercial gypsum) further enhanced the UCS by more than 100% compared to recycled gypsum and increased the cohesion (c’) values from 0 to 32.8–47.2 kPa. The compression index (c_c_) decreased from 0.15 to 0.05, and the maximum volumetric strain (ε_v_) at an applied effective stress of 800 kPa decreased from 17% to 5%. Emulsified polymer treatments also enhanced the mechanical and hydraulic properties of the clayey tailings; however, the overall improvements were lower than those achieved with CKD–gypsum blends, suggesting that further optimization of the polymer concentration or its combination with mineral additives may yield better results. These findings offer a foundation for further research into the use of polymers in geoenvironmental applications, particularly for erosion control, contaminant encapsulation, and hydraulic barrier development. Overall, this study highlights the potential of using industrial by-products, such as CKD and gypsum, as sustainable, cost-effective materials to improve tailing performance, while identifying promising directions for polymer-based solutions in geotechnical engineering.

## 1. Introduction

The extraction of valuable minerals often produces fine-grained residues known as mine tailings (MTs). For every ton of valuable mineral extracted, up to 99% of the processed material may become mine tailings, depending on the ore grade, extraction process, and ore material [1]. Significant environmental and geotechnical challenges arise from MTs due to several factors—most importantly, their massive quantities, the chemical and mechanical processes the extracted rocks undergo before ore is produced, and the consequent environmental problems, such as oxidation/leaching and water infiltration. Annually, a single mine can generate between 1 and 6 million cubic meters of tailings.

Being of low economic value, the MT materials are typically stored in natural or manmade tailings storage facilities (TSFs). Manmade TSFs are commonly constructed using upstream, downstream, or centerline methods [2,3]. Owing to their low costs, upstream-constructed earth dams are often used [4]. However, because these tailings dams rely on tailing materials for construction, they are particularly vulnerable to slope instability [5]. Upstream dams have a well-documented history of failures triggered by static liquefaction, elevated pore pressures, a rapid drawdown, seismic loading, and inadequate drainage [6,7,8,9]. Such failures can result in catastrophic environmental and human losses, as demonstrated by the Brumadinho dam collapse in Brazil (2019), which resulted in over 270 fatalities and significant contamination. Beyond humanitarian and environmental impacts, tailing dam failures can disrupt global commodity markets, as seen after the Brent Rodriguez failure, when iron prices rose by more than 20%. Other notable failures, including those of El Cobre (Chile, 1965), Shanxi Province (China, 2008), and Buffalo Creek (USA, 1967), have collectively caused over 550 fatalities [7,8,9,10,11]. In response to these events, regulatory frameworks have evolved. Organizations such as the Canadian Dam Association (CDA) and the Mining Association of Canada (MAC) now emphasize comprehensive risk assessments that consider extreme loading and long-term tailing behavior [12,13]. Despite these advances, tailing stabilization remains a major engineering challenge, driving the need for innovative and sustainable solutions.

**Figure 1:**
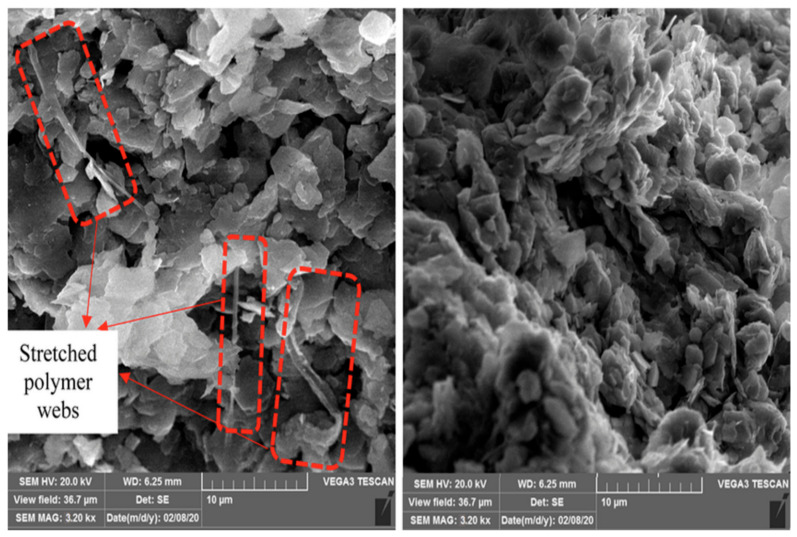
Polymer-improved soil SEM image, revealing particle bonding.

To overcome these challenging conditions, ground improvement techniques can be used. Ground improvement techniques can be grouped into mechanical, chemical, and rigid inclusion methods. Mechanical approaches include densification and consolidation techniques such as compaction, soil replacement, preloading, and dynamic methods. These approaches are typically applied to cohesionless materials and are often used on outer dam slopes, where the relative density must exceed 95% [14,15]. When mechanical methods are insufficient, chemical stabilization—using lime, cement, deep soil mixing, or grouting—is employed to improve strength and reduce settlement [16,17,18]. More specialized applications may require rigid inclusions such as stone columns, geogrids, or piles [19,20,21], or advanced techniques such as deep soil mixing and geocomposites [22,23,24,25,26,27,28,29,30,31]. Prior research has demonstrated that additives such as geopolymers significantly improve erosion resistance, reduce swelling in expansive soils [20] and offer faster strength development compared to conventional cementitious binders, Figure 1 shows a scanning electron microscopy (SEM) image of polymer-improved soil, highlighting the formation of polymer films and particle bonding. These microstructural features support the macroscopic behavior observed in the experimental results, particularly the increase in apparent cohesion and changes in compressibility. Separately, CKD–bassanite (CKD:B) combinations were evaluated at dosages of 5%, 10%, and 20% by dry weight to assess their contribution to strength gain through pozzolanic activity and flocculation.

Industrial by-products such as cement kiln dust (CKD) and recycled gypsum have also demonstrated potential as sustainable soil stabilizers [23,24]. CKD, which is rich in calcium oxide, promotes stabilization through flocculation and chemical bonding [32]. Recycled gypsum, often processed as bassanite (CaSO_4_·½H_2_O), can further enhance strength and durability through secondary cementitious reactions when activated by CKD [33].

Despite the growing interest in polymer-based soil stabilization, limited research has systematically compared polymer-based and mineral-based stabilization approaches for tailings, particularly in terms of their combined mechanical and hydraulic performance. Furthermore, the interaction between tailings and emulsified acrylic polymers (EAPs) and industrial by-products such as cement kiln dust (CKD) and gypsum remains insufficiently understood. This study provides a comparative experimental evaluation of polymer-based and mineral-based stabilization approaches, focusing on the combined effects of emulsified acrylic polymer (EAP), CKD, and gypsum on the mechanical and hydraulic behavior of tailings. The objectives were to: (1) characterize the geotechnical and chemical properties of untreated tailings and (2) assess the effects of emulsified acrylic polymer (EAP)–CKD–gypsum stabilization on strength, compressibility, and hydraulic behavior. This research provides experimental and computational evidence supporting the use of emulsified acrylic polymer (EAP)- and CKD-based additives as effective, safer, and more sustainable solutions for tailing management.

## 2. Methodology

**Figure 2:**
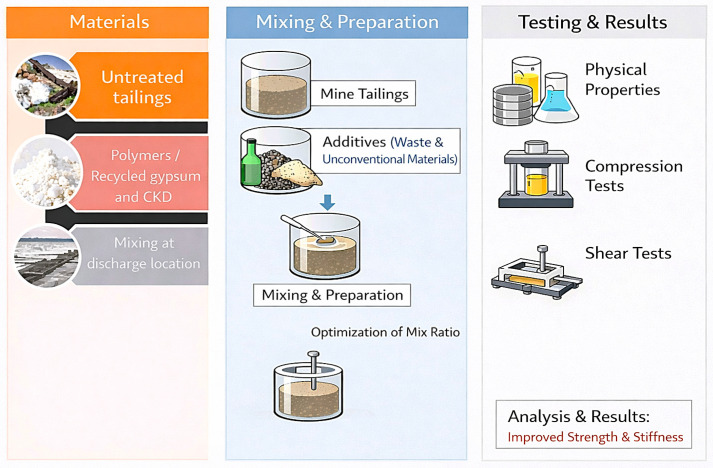
Proposed treatment of the tailing stabilization study.

**Figure 3:**
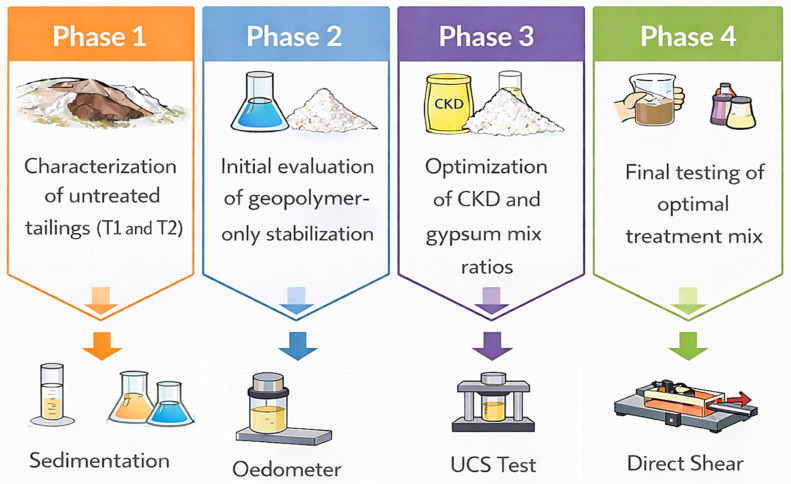
The experimental program phases adopted in the study.

A structured experimental program was conducted to investigate the effects of different additives on tailing behavior under compression and shear. The methodology was designed to enable direct comparison between polymer-based and mineral-based stabilization approaches using consistent preparation and testing procedures. The procedure involved determining the physical properties of the tailings and admixtures, followed by controlled mixing to improve stiffness and strength. The untreated tailings were prepared from slurry and dewatered prior to specimen preparation. The initial water content used for oedometer specimen preparation was slightly higher than the liquid limit, with a measured value of 51.1%, while the corresponding dry unit weight was determined after specimen preparation and testing. The additives were introduced gradually into the tailing slurry and mixed either manually or with a hand mixer for approximately 5 min to ensure uniform dispersion and homogeneity. After casting, the specimens were left under laboratory conditions at ambient room temperature (20–22 °C) for initial setting and were subsequently cured for 7 or 14 days. Figure 2 and Figure 3 show the proposed treatment methodology. All tests, as well as the analysis of the tailings’ physical properties, were conducted in accordance with the relevant ASTM standards.

This section outlines the experimental program to investigate the stabilization of mine tailings using a ternary additive system comprising emulsified polymer, CKD, and recycled gypsum. The experimental investigation involved material characterization, development of the treatment matrix, sample preparation, and a detailed laboratory testing program.

### 2.1. Experimental Program Overview

The experimental investigation was structured into four main phases (see Figure 3):

1. Characterization of untreated tailings (T1 and T2), where T1 represents silty tailings (ML–SMs) and T2 represents low-plasticity clayey tailings (CLs), used at different stages of the experimental program.
2. Initial evaluation of EAP-only stabilization.
3. Optimization of CKD and gypsum mix ratios through UCS.
4. Final testing of optimal treatment mix.

Each phase included the following specific geotechnical tests: an unconfined compressive strength (UCS) test, direct shear tests, oedometer consolidation tests, sedimentation studies, and chemical analysis.

### 2.2. Materials

Two tailing types were sampled from decommissioned mines in Northern Ontario:

- Tailing 1 (T1): Classified as silty sand (ML-SM) with 68% silt.
- Tailing 2 (T2): Classified as clay with low plasticity (CL).

The tailings were characterized by using a suite of geotechnical and chemical tests, as reported by [1]. The characterized properties included the particle size distribution (sieve and hydrometer methods), Atterberg limits, specific gravity, moisture content, and chemical composition assessed through inductively coupled plasma (ICP) and X-ray fluorescence (XRF) spectroscopy.

**Figure 4:**
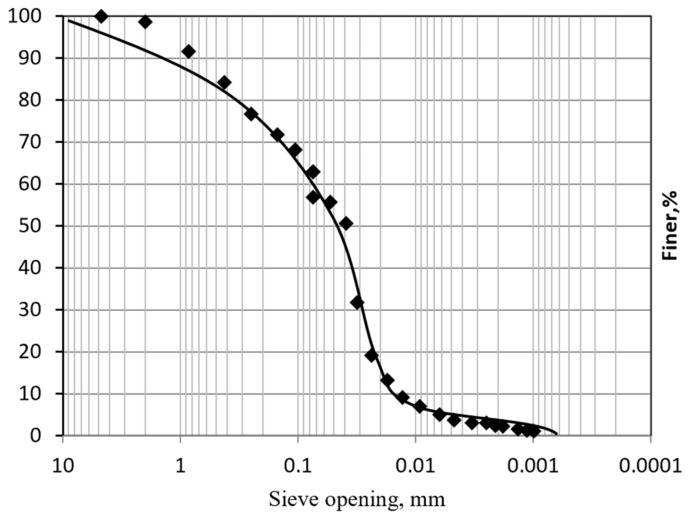
Sieve analysis test and hydrometer test graph for tailings.

ASTM (ASTM 2487) [34] and the Unified Soil Classification System (USCS) classified the soil as silt. The results shown in Figure 4 and Table 1 detail the mean particle size, D_50_%; effective particle size, D_10_%; the coefficient of uniformity (C_u_); and the coefficient of curvature (Cc). The oven-dried tailings were sieved through a #40 U.S. sieve, disintegrated carefully, and tested for the liquid limit (LL), plastic limit (PL), and plasticity index (PI) using Casagrande’s apparatus (ASTM D4318) [35]. The tailings were classified as low-plasticity silt, ML.

**Figure 5:**
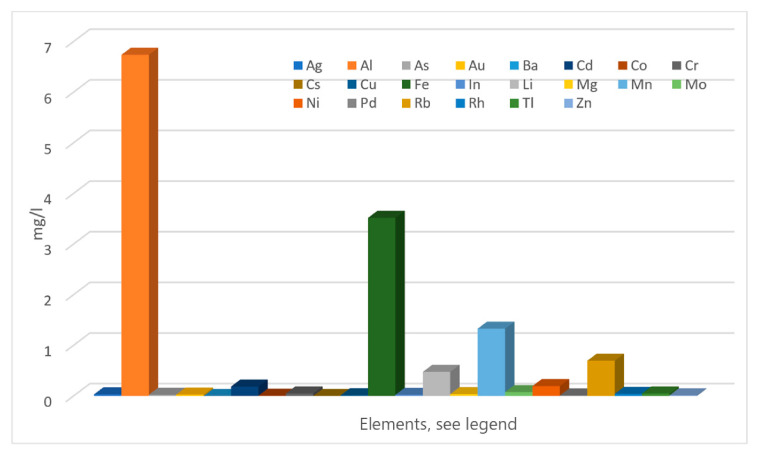
Chemical analysis of T1 water.

Chemical analyses (Table 2) revealed that all tested tailing materials were rich in calcium, with plaster exhibiting the highest concentration (67.4%), followed by recycled gypsum (53.7%). These values suggest a higher potential for strength development in treated soils. Both plaster and recycled gypsum also showed elevated sulfur trioxide levels, which may pose environmental risks due to the contribution of sulfur trioxide to acid rain formation. The tailings were found to be lightweight in composition, as indicated by a relatively high silicon and aluminum oxide content and a specific gravity of 2.69—characteristic of silty clay soils (Table 1). Unlike some tailings that exhibited elevated levels of heavy metals, the solid-phase samples in this study showed no such anomalies. However, the plaster did contain arsenic at a concentration of 0.024 g/kg, surpassing the Canadian Drinking Water Guideline limit of 0.01 mg/L. Additionally, hazardous elements detected in the tailing liquid—such as arsenic, cadmium, chromium, and lead—exceeded the guideline limits by factors of 2.4, 37.5, 1.0, and 1.06, respectively (see Figure 5). These results highlight the urgent need for robust lining and filtration systems in tailings storage facilities (TSFs) to prevent contamination through leakage or seepage. Historical failures, such as the cyanide-laden tailings spill in Romania [6], underscore the critical importance of TSF integrity. To reduce environmental risk, particularly from acid mine drainage (AMD), tailings are often kept submerged to limit their exposure to oxygen and suppress oxidation reactions.

#### 2.2.1. Chemical Soil Stabilization

Chemical soil stabilization techniques were used to improve the tailings; this involved the use of conventional and unconventional additives. The additives used are listed below:

- Emulsified acrylic polymer (EAP): An anionic emulsified acrylic polymer (EAP) with 47% solids, a low viscosity (13 mPa.s), and quick-setting characteristics.
- Cement kiln dust (CKD): An industrial by-product rich in calcium hydroxide.
- Recycled gypsum (B): Sourced from construction waste and processed into bassanite.

This study investigated the use of both traditional and non-traditional additives to improve the strength and stiffness of tailings, particularly those used in upstream tailings impoundments. Traditional additives, such as lime, cement, fly ash, gypsum, cement kiln dust (CKD), and slag, are well-established in geotechnical practice. In contrast, non-traditional additives—including polymers, fibers, geotextiles, and EAP—represent more recent innovations in soil stabilization. Table 3 and Table 4 present the basic information of the EAP product used in this study. The product comes in 4 components (Table 3) which are mixed together to produce the final EAP product (Table 4).

To comparatively evaluate the performance and identify the most effective binder, traditional additives—specifically recycled gypsum (B) and cement kiln dust (CKD)—were also tested. These were mixed with tailings (provided by Golder Associates) at varying ratios and total contents of 5%, 10%, and 20% by weight. Recycled gypsum was sourced from Rona, and CKD from a commercial supplier. The tests were conducted after curing periods of 7 and 14 days to evaluate early-stage strength development. While longer curing durations (e.g., 28 days) are commonly used for cementitious systems, the focus of this study was on early performance relevant to staged construction in tailings dams. The potential for further strength gain at later curing stages is acknowledged and recommended for future investigation. The study objectives were: (1) to assess the effect of increasing the gypsum content in the blend and (2) to explore the cost-reduction potential through the use of recycled materials. The optimal B-to-CKD blend was selected for further application in tailing stabilization. Table 5 presents the combination used herein.

#### 2.2.2. Mix Design and Optimization

Initially, the EAP product was evaluated independently at dosages of 0.5%, 1%, and 2% by dry weight using T1. This stage was intended as a preliminary screening study to establish the baseline response of the polymer-treated tailings before pursuing more complex multi-factor designs. Subsequently, CKD and recycled gypsum were blended at ratios of 1:1, 1:2, and 1:4 and incorporated into T2 at increasing additive contents. An optimization matrix was then developed (see Table 5) to compare the different treatment approaches on the basis of UCS, compressibility, and hydraulic behavior. The optimal mix was selected for extended testing using oedometer and direct shear tests. The polymer phase is therefore presented as an initial comparative assessment rather than a full parametric optimization study.

### 2.3. Sample Preparation and Testing

**Figure 6:**
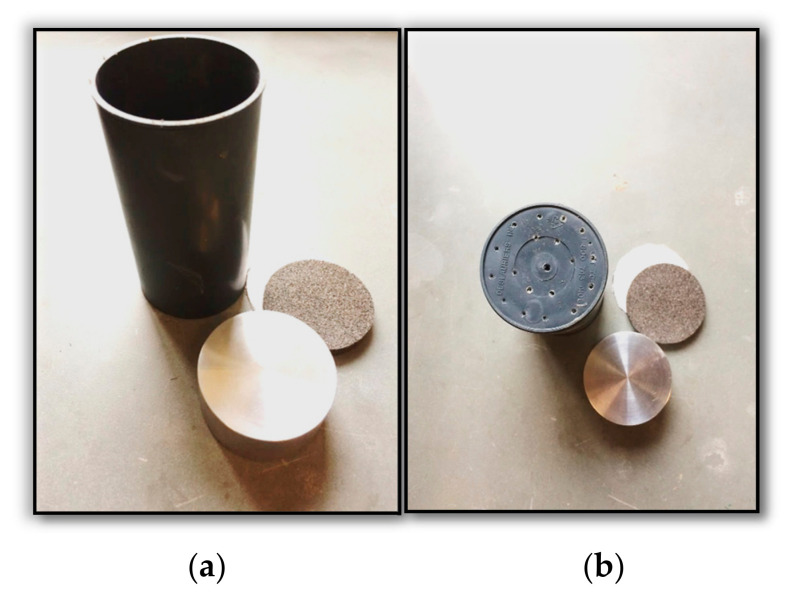
(**a**) The tools used for the dewatering setup; (**b**) perforation to enable water to be collected from the bottom.

Figure 6 shows the setup used to dewater the slurry to a zero-effective-stress void ratio. The setup comprised a plastic cylinder, a porous stone, and filter paper.

The boiled porous stone and filter paper were used to seal the bottom to ensure that water exited from the perforations when the viscous tailings were squeezed. The load was applied for 24 h based on the soil behavior observed during the sedimentation column, where the excess pore water pressure dissipated and the soil consolidated within one day. After 24 h of dewatering, the soil was transferred to the oedometer ring, the W_c_% was measured, and the sample was tested in the oedometer according to ASTM D2435 [36]. The sample was allowed to consolidate in the oedometer for 12 h. The initial water content was slightly higher than LL, at 51.1%, which demonstrates the effectiveness of the dewatering technique used before starting the consolidation test. Measurements of the coefficient of consolidation, c_v_, were taken for the untreated tailings at different loading steps to establish the lower and upper bounds. Cylindrical specimens were then compacted into molds measuring 50 mm × 100 mm for consolidation testing and later in a rectangular shear box measuring 38 mm × 76 mm for direct shear tests (DSTs). For EAP-treated specimens, the hardener and accelerator components were added immediately prior to mixing. EAP batches were blended using a kneader for 5 min to ensure a uniform consistency and were then poured into molds. All samples were cured at ambient temperature (20–22 °C) for 7 and 14 days. The oedometer samples were cast using molds designed with bottom drainage, incorporating filter paper and porous stone to facilitate consolidation. The test procedure followed ASTM D2435, and the data was used to determine the compression index (c_c_), coefficient of consolidation (c_v_) based on Taylor’s method, and the hydraulic conductivity (k).

Oedometer testing was selected to assess the effectiveness of the EAP for soil stabilization. The emulsion used in this study formed a rubber-like matrix upon curing and, according to the supplier, achieved a full strength of approximately 1 MPa within 24 h. This method was chosen for its efficient sample preparation, which minimizes disturbance and preserves the integrity of the material properties. Furthermore, oedometer testing provides critical geotechnical parameters—including hydraulic conductivity, constrained modulus of elasticity, and c_v_—essential inputs for modeling soil behavior using the Mohr–Coulomb constitutive framework.

**Figure 7:**
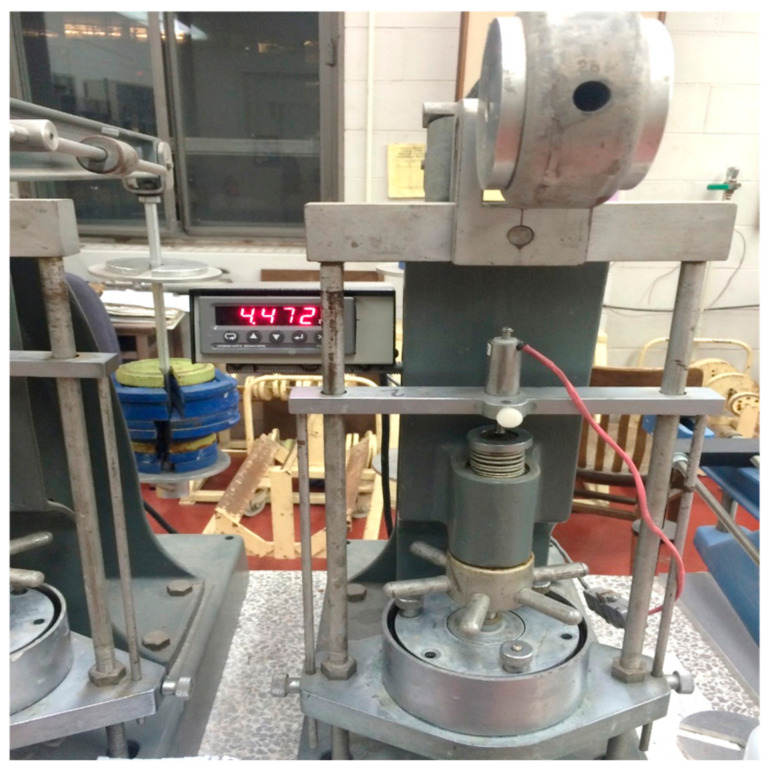
Setup of an oedometer test showing container and LVDT attachment.

After 24 h of initial curing, the treated tailings specimens were carefully demolded and placed into consolidation rings. Care was taken to avoid remolding; minimal manual adjustment and light settling were used to allow the sample to conform to the ring shape naturally. The specimen surfaces were then leveled to ensure the proper alignment and seating of the loading cap. The assembled oedometer units were positioned in the loading frame, and testing was performed in accordance with ASTM 2435 standards. Figure 7 shows the test setup. The compression index (c_c_) derived from this test indicates the compressibility behavior of the soil. To ensure consistency, c_c_ values for the treated and untreated samples were calculated over comparable effective stress intervals:

(1) $$c_{c}=\frac{e_{1}-e_{2}}{log(\frac{\sigma_{2}^{\prime }}{\sigma_{1}^{\prime }})}$$

Here, e represents the sample’s void ratio while σ’ denotes the corresponding effective stress; subscripts 1 and 2 are any given points on the virgin consolidation line.

The typical values of c_c_ range from 0.1 to 0.2 for silty soils and from 0.2 to 0.3 for clayey soils [10]. No rebound measurements were recorded for the untreated materials. Therefore, only the c_s_ values were compared to the treated tailings.

**Figure 8:**
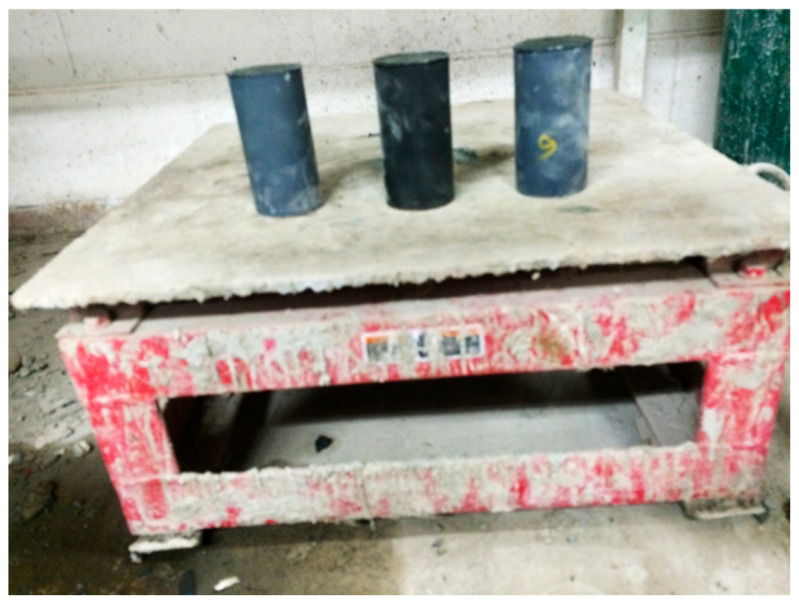
Mixed tailings are placed on the shaking table.

Unconfined compressive strength (UCS) tests were performed in accordance with ASTM D2166 on tailings specimens treated with varying dosages of CKD:B (cement kiln dust to bassanite) mixtures at 5%, 10%, and 20% by dry weight of the tailings (see Figure 7). The additives were gradually incorporated into the tailings slurry while continuously mixing with a hand mixer to ensure uniform dispersion and homogeneity. It is well-established that traditional cementitious admixtures typically achieve most of their strength gain within the first 28 days of curing [37]. In contrast, gypsum-treated soils often reach peak strength within the first 14 days [33], making early strength development a key consideration in this study. Following a 5 min mixing period, the blended slurry was cast into greased cylindrical molds in three equal layers. Each layer was compacted by tapping 10 times with a 2 mm diameter rod to eliminate air voids and ensure a consistent density. The fully assembled molds—50 mm in diameter and 100 mm in total height—were then placed on a shaking table for 2 min to enhance consolidation further and remove entrapped air, as illustrated in Figure 8.

**Figure 9:**
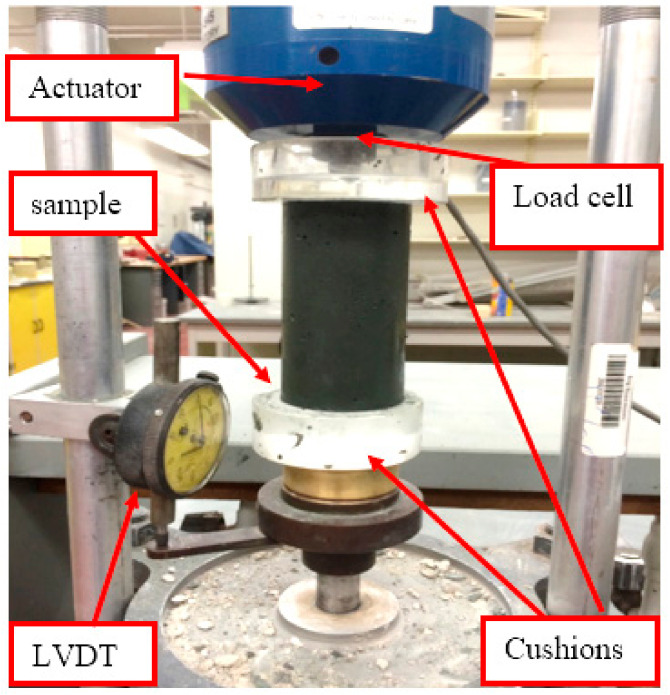
UCS sample setup. The device was equipped with a dial gauge to measure the vertical displacement.

Subsequently, the cast samples were left exposed to ambient laboratory air at 20–22 °C for 24 h to allow for initial setting, after which they were capped with lids and cured for periods of 7 and 14 days. The laboratory relative humidity was actively controlled at 95%, and all specimens were prepared and cured under the same indoor environmental conditions to ensure consistency between treatment groups. The UCS tests were performed using a strain-controlled machine at a rate of 4.3 mm/min. Given that gypsum-treated soil typically attains its maximum strength within the first 14 days, the tests were conducted after curing periods of 7 and 14 days. A total of 30 samples were prepared, with half tested after 7 days and the remaining half after 14 days of curing. After the specified curing periods, the samples underwent UCS testing. It is important to note that, in many of the sheared samples, the failure plane was not distinctly defined. Figure 9 illustrates the final sample setup before testing.

### 2.4. Direct Shear Test (DST)

To establish the shear strength parameters required for subsequent finite element modeling, direct shear tests (DSTs) were conducted on both the treated and untreated tailings using the optimal treatment formulations. These tests were aimed at deriving the Mohr–Coulomb constitutive parameters under conditions that reflect the anticipated field behavior. Since tailings in the field are neither fully drained nor fully undrained due to variable gradation and dissipation of pore pressure—especially with increasing distance from the discharge point—an effective stress analysis (ESA) was adopted. The ESA allowed the distribution of excess pore pressure (EPP) along potential slip surfaces to be incorporated into the numerical model. The direct shear device used in this study featured a 60 mm × 60 mm square shear box and a fully computerized data acquisition system operated on DaisyLab graphics user interface (GUI). Displacement measurements were captured using two high-precision linear variable differential transformers (LVDTs) with an accuracy of ±0.01 mm, and the data were logged using DaisyLab. The apparatus was equipped with two loading cells—one vertical and the other offset to enable mechanical loading via a lever-arm mechanism. The maximum normal stress capacity of the system was 0.6 MPa. Tests were conducted in accordance with ASTM D3080 [38], using strain-controlled loading at a rate of 0.03 mm/min.

## 3. Results

### 3.1. Conventional Binder Optimization Study

Additives were incorporated into the tailings at concentrations of 5%, 10%, and 20% by total dry weight. The prepared mixtures were cast into plastic molds and subjected to unconfined compressive strength (UCS) testing after curing periods of 7 and 14 days. The objective of this phase was to evaluate the effect of increasing the recycled gypsum content on the mechanical performance of the mixtures and to identify the optimal B:CKD (recycled gypsum to cement kiln dust) ratios for further testing.

**Figure 10:**
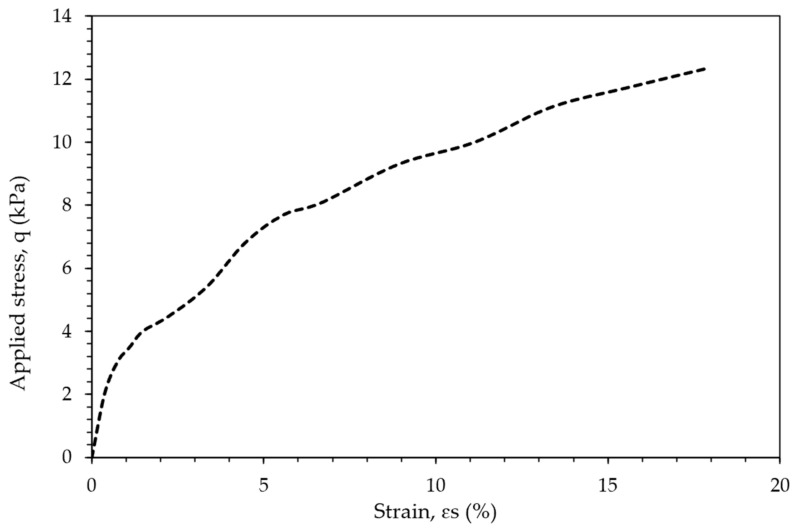
Stress–strain relationship of tailing 2 treated with 1CKD:2B at 10% obtained from the UCS tests.

**Figure 11:**
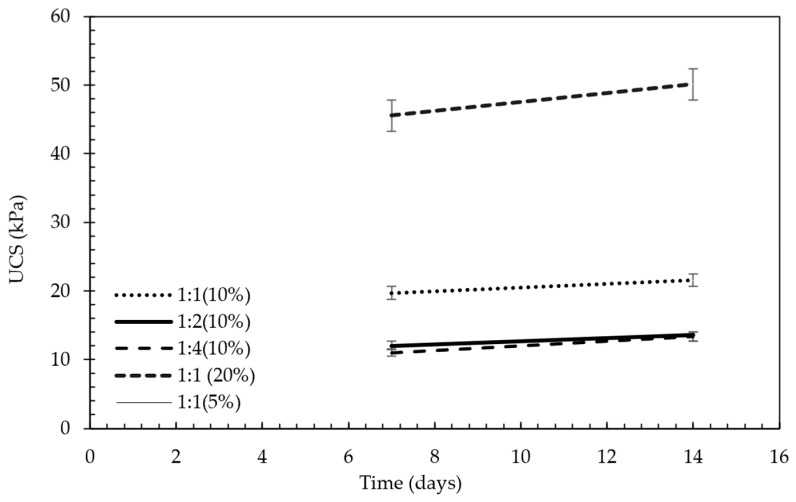
UCS of the five combinations for the period of testing.

**Figure 12:**
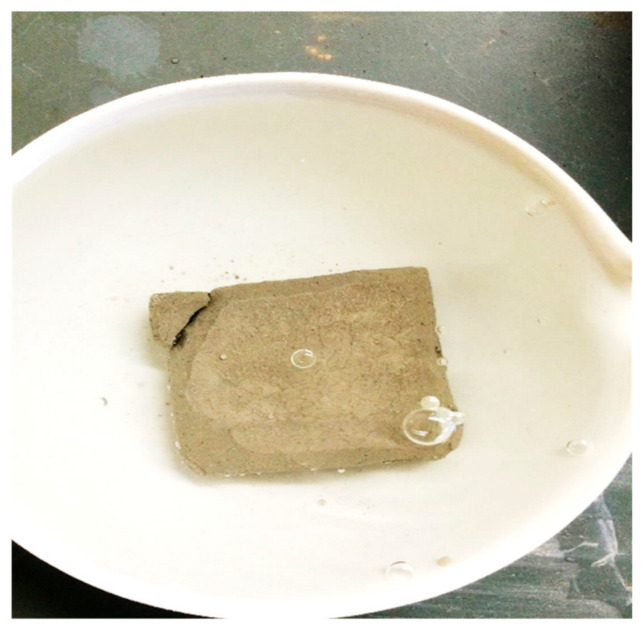
DST sample with CKD:B and OC dispersion test.

Following this evaluation, the most effective B:CKD ratios were determined and consistently used in subsequent stages of the study. Figure 10 presents a representative stress–strain curve from the UCS tests, while Figure 11 summarizes the optimization analysis, from which the optimal additive percentages were derived for comparison with the EAP-based treatments. It is important to note that when the samples were soaked in water for consolidation testing, no improvement in strength was observed. This behavior was attributed to the dissolution of soluble phases and the associated breakdown of interparticle bonding under saturated conditions. To mitigate sample disintegration during subsequent testing, a small amount of ordinary Portland cement (OC) was introduced as a supplementary stabilizing component. The role of OC in this study was limited to preserving specimen integrity during water exposure, and its dosage was selected on the basis of preliminary dispersion behavior rather than a full optimization program. Accordingly, the interaction between OC and the CKD–gypsum system is acknowledged but was not investigated as an independent research variable in the present work. The effectiveness of these additions was evaluated using a dispersion test (see Figure 12), which helped identify the minimum OC dosage required to maintain structural integrity under saturated conditions.

### 3.2. Oedometer Results

**Figure 13:**
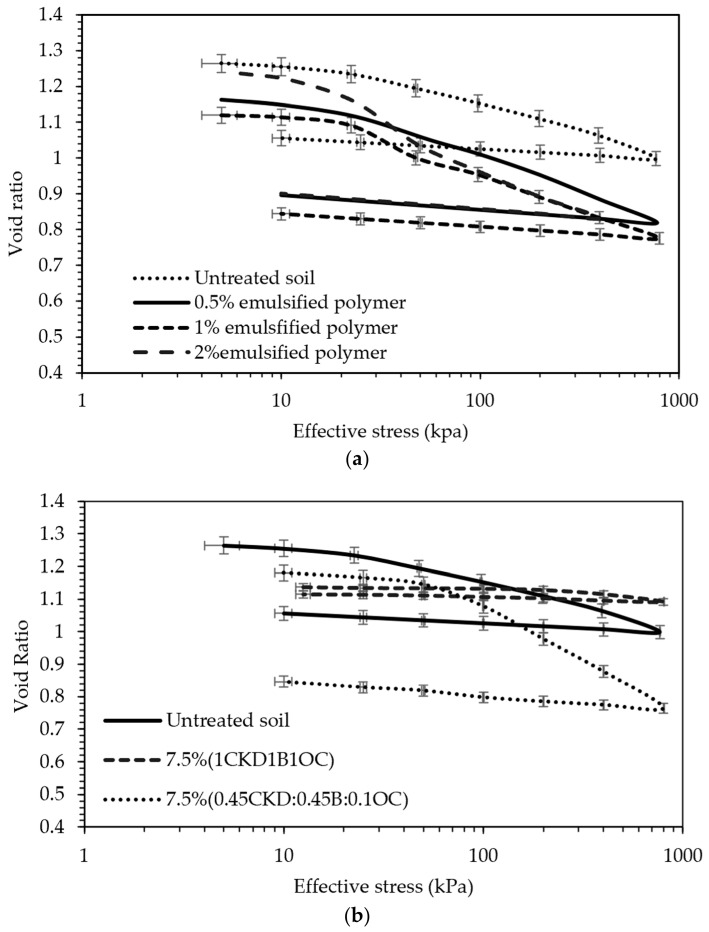
Void ratio versus logarithmic effective stress plots for the various tested additive percentages; (**a**) polymeric treatment and (**b**) CKD:OC:B treatment.

Figure 13 presents the e–log σ′ (void ratio vs. effective stress) curves derived from the consolidation tests for both treatment strategies. The incorporation of the EAP increased the overall solid content of the tailings matrix, thereby reducing the void ratio. This effect mimics a dewatering process—not through the physical removal of water, but by increasing the solid-to-liquid ratio, thereby lowering the volumetric water content. Consequently, the dry unit weight of the treated samples increased. However, despite the denser matrix, the EAP treatment led to an increase in soil compressibility, as shown in Figure 13a. For untreated soil, the compression index was 0.1548; for tailings treated with 0.5, 1, or 2% polymer, c_c_ was 0.2265, 0.1928, and 0.2302, respectively. These results indicate that the untreated tailings exhibited greater stiffness than the treated samples. The slightly improved response observed at 1% dosage may be attributed to localized variations in bonding and material distribution than the 0.5% and 2%. It should be noted that these percentages were chosen because they have been used in the literature and for economic reasons [39,40]. In contrast, the CKD:B:OC treatment enhanced interparticle bonding in the tailings, thereby improving strength and stiffness. This was reflected by a flattered consolidation curve and a lower compression index, indicating a reduced compressibility and greater resistance to deformation under load (Figure 13b).

**Figure 14:**
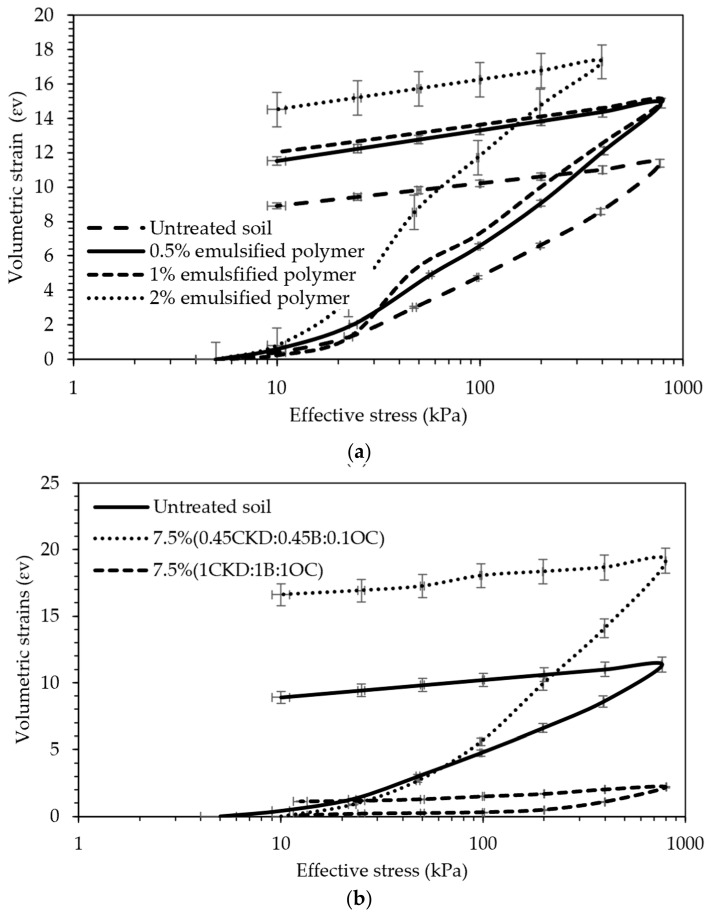
Volumetric strain–logarithmic effective stress relationship: (**a**) polymer treatment and (**b**) CKD:B:OC treatment.

Figure 14 illustrates the relationship between volumetric strain and logarithmic effective stress for the tested specimens. Strain measurements were recorded before unloading the initial 5 kPa seating pressure to better capture the compressibility behavior of the treated tailings under applied stress conditions. As shown in Figure 14a, the addition of EAP did not significantly enhance the stiffness of the tailings, despite yielding a higher dry unit weight. This observation can be attributed to the rubber-like characteristics of the EAP; although this increases the solid content, it replaces stiffer soil particles with a more deformable matrix, resulting in a limited resistance to compression. In contrast, Figure 14b demonstrates a substantial reduction in compressibility for samples treated with the CKD:B:OC (ordinary Portland cement) blend. When employing a 7.5% admixture ratio of 0.45CKD:0.45B:0.1OC, the tailings stiffness did not exhibit a significant increase. This could be attributed to either weak or insufficiently formed bonds, leading to bond collapse when the effective stress exceeds 50 kPa. Conversely, a notable enhancement was observed in tailings treated with a 7.5% admixture of 1CD:1B:1OC. Volumetric strains notably decreased from 17% to 2%, as depicted in Figure 14b. The inclusion of CKD and gypsum, along with small cement additions, improved interparticle bonding and structural integrity, thereby enhancing stiffness and reducing volumetric strain under loading.

For the final treatment plan obtained from the initial UCS program, a minimum of three specimens were prepared and tested for each condition to ensure repeatability and reduce experimental variability and tested at three different normal stress levels to construct the final shear strength envelopes. The shear box dimensions met laboratory standards: the ratio of the shear box’s internal dimensions to the maximum particle size was 30, exceeding the minimum standard of 10, and the thickness-to-particle size ratio was 20, exceeding the required minimum of 6 based on ASTM D3080.

Untreated tailings generally exhibit low shear strength, often characterized by negligible or very low apparent cohesion and relatively low friction angles, particularly in fine-grained or loosely deposited materials. Previous studies have reported cohesion values approaching zero for silty tailings and friction angles typically ranging from approximately 20° (for clayey sized tailings where the behavior is undrained) to 35° under low-density or undrained conditions, although higher values may be observed depending on density and particle composition [41,42,43]. It should be noted that these values correspond to post peak shear strength obtained from individual tests. In contrast, the parameters presented in Table 6 are derived from regression of the Mohr–Coulomb failure envelope. This distinction has been clarified to ensure consistency in data interpretation. In contrast, the treated specimens—stabilized with a blend of 7.5% OC:CKD:B (at a 1:1:1 ratio) showed substantial improvement. The cohesion values increased from 0 to 180–203 kPa, and the friction angles ranged from 38 to 40°, depending on the specific tailings type. These values are consistent with those reported in the literature for polymer-modified clays and cementitious soil blends [44,45,46,47,48].

## 4. Discussion

This section discusses the results of the geotechnical and physical characterization of tailings treated with various additives. Although direct microstructural analyses, such as scanning electron microscopy (SEM) or Fourier-transform infrared spectroscopy (FTIR), were not performed in this study, the observed increase in the compressibility of emulsified acrylic polymer (EAP)-treated tailings can be interpreted in light of findings from prior microstructural investigations.

Previous studies have shown that emulsified polymers form flexible films or coatings around soil particles as shown in Figure 1, resulting in increased apparent cohesion while introducing deformable zones within the soil matrix. This microstructural behavior is consistent with the experimental observations in this study, where polymer-treated tailings exhibited increased cohesion but also higher compressibility. In contrast, CKD–gypsum treatments promote more rigid interparticle bonding, which explains the observed improvement in stiffness and reduction in volumetric strain [13,27]. SEM observations reported in the literature indicate that, unlike cement-treated soils, which develop rigid interparticle bridges and crystalline bonding structures, EAP-treated soils typically exhibit smooth surfaces with fewer crystalline connections [42,46,47,48].

These microstructural characteristics led to a reduction in permeability while simultaneously enhancing compressibility under applied loads—an outcome consistent with the oedometer test results obtained in this study. This behavior underscores the dual nature of the EAP treatment: while it improves certain mechanical properties such as cohesion and erosion resistance, it may also increase the material’s susceptibility to volumetric strain under stress.

### 4.1. Oedometer Tests

The primary objective of the emulsified acrylic polymer (EAP) treatment was to reduce the tailings’ compressibility, as indicated by a lower compression index (c_c_). However, the results showed that the untreated tailings had a c_c_ of 0.16, while treatment with 0.5%, 1%, or 2% EAP resulted in increased values of 0.2265, 0.1928, and 0.2302, respectively, representing increases of approximately 46%, 25%, and 49% relative to the untreated condition. Although the 1% EAP-treated sample exhibited a marginally lower c_c_ than the other treated samples, all treated samples showed a clear trend toward increased compressibility. These findings indicate that, contrary to expectations, the addition of this particular EAP softened the tailings rather than stiffening them.

This counterproductive effect is attributed to the polymer’s rubber-like nature, which, while increasing the dry unit weight, introduces a more compressible matrix than the original soil particles. The selected polymer contents of 0.5%, 1%, and 2% by weight were chosen as a practical screening range based on prior literature and economic considerations. The purpose of this phase was not to identify a globally optimum polymer formulation, but rather to determine whether the tested EAP showed sufficient promise to justify more advanced multi-variable investigation. Because the polymer-treated specimens did not exhibit favorable compressibility behavior, further optimization of polymer dosage or combined polymer–mineral systems was not pursued within the scope of the present study. Despite their low mass percentages, the EAP’s liquid state and water-like unit weight meant that these additions significantly increased the total volume, affecting the soil structure and void distribution.

In contrast, tailings treated with a blend of cement kiln dust (CKD) and recycled gypsum (denoted by CKD:B) showed a favorable response. Although initial values were only in ten of kPa, these values present significant improvement due to the tailings being mixed as a slurry with essentially zero effective stress and shear strength properties. These treatments reduced the compression index to a range of 0.06–0.1, roughly a 40–60% decrease compared to untreated tailings (c_c_ ~0.16–0.2), and improved the coefficient of consolidation (c_v_) to approximately 3.6 × 10^−8^ m^2^/s—nearly three times higher than that of the untreated sample (c_v_ ~1.3 × 10^−8^ m^2^/s). Moreover, the initial hydraulic conductivity (k) of untreated tailings was on the order of 1 × 10^−7^ m/s, whereas stabilization reduced k by over an order of magnitude (1.2–2.0 × 10^−8^ m/s), primarily due to densification of the matrix and the decreased pore continuity induced by emulsified acrylic polymer (EAP) encapsulation mechanisms.

Overall, the oedometer test results demonstrate distinct differences between polymer-based and mineral-based stabilization mechanisms. While the emulsified acrylic polymer increased apparent cohesion, it also introduced a more deformable matrix, leading to increased compressibility. In contrast, CKD–gypsum treatments resulted in stronger interparticle bonding and reduced compressibility, indicating a more effective stabilization mechanism for structural applications. This highlights that not all polymeric additives enhance mechanical performance, and careful material selection is crucial for effective tailing stabilization. Consequently, further investigation into this specific EAP was discontinued in the current study.

### 4.2. UCS

**Figure 15:**
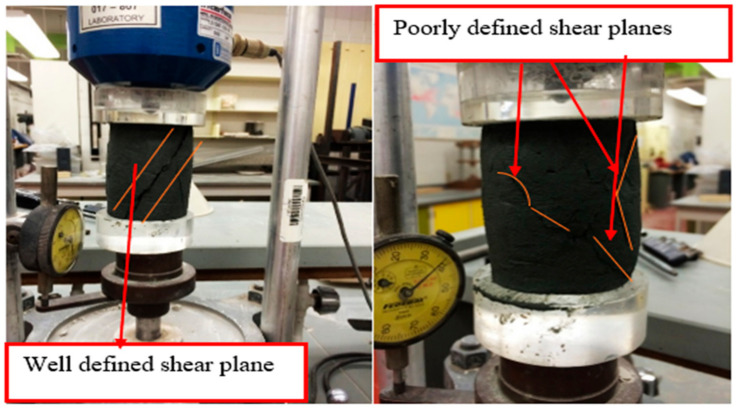
Shearing planes of untreated (**left**) and treated (**right**) tailings sample.

Throughout all 30 UCS tests, shearing was conducted over a duration of 240 s. Figure 15 depicts a representative sample following the completion of testing for both treated and untreated tailings samples. As shown in Figure 15, the failure plane was not clearly defined for the sheared treated tailings samples, unlike the untreated samples. Following the completion of shearing, water content measurements were taken to determine the dry unit weight. It was observed that the dry unit weight increased with higher concentrations of additives, which was attributed to three reasons: (1) their filler effects, (2) their chemical reaction and hardening process, and (3) dehydration effects.

#### 4.2.1. Effects of Internal Forces on the Treated Soil

Under undrained conditions in unconsolidated clay samples, the maximum undrained shear strength is theoretically independent of the principal stresses (σ_1_ and σ_3_). Accordingly, applying confining pressure without allowing consolidation should, in theory, not alter the undrained strength. However, under real-world conditions, particularly where vertical stress and settlement evolve over time, it is critical to understand how these changes affect the treated material’s mechanical behavior.

Environmental factors, such as wetting–drying cycles, can induce volumetric changes that alter the soil’s internal structure, potentially affecting its strength and stiffness. Similarly, in regions susceptible to freeze–thaw events, repeated freezing and thawing may degrade interparticle bonds within the stabilized matrix, undermining its long-term performance. These influences must be anticipated during the design phase to prevent discrepancies between expected and in situ behavior.

It is important to note that this study did not investigate the effects of wet–dry cycles or freeze–thaw cycles, both of which are recognized as important durability factors for stabilized tailings in field applications. Instead, the present work focused on short-term mechanical and hydraulic performance under controlled laboratory conditions. One-dimensional consolidation tests were performed up to a maximum vertical stress of 800 kPa—within the typical operational stress range for mine tailings embankments—to assess whether elevated or cumulative stresses could lead to bond degradation. The durability of the proposed treatment system under repeated environmental cycling remains an important topic for future study. However, one-dimensional consolidation tests were performed up to a maximum vertical stress of 800 kPa—within the typical operational stress range for mine tailings embankments (0–2 MPa)—to assess whether elevated or cumulative stresses could lead to bond degradation. The implications of this loading regime will be further discussed in subsequent sections.

#### 4.2.2. Effects of Curing Days

**Figure 16:**
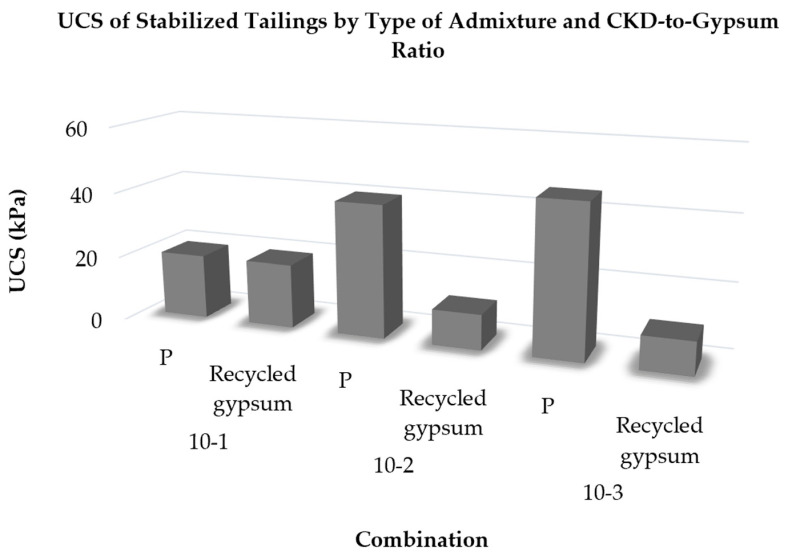
Variation in unconfined compressive strength (UCS) of tailings stabilized with 10% admixture at different CKD-to-gypsum ratios, comparing commercial plaster with recycled gypsum.

Figure 16 depicts the evolution of the unconfined compressive strength of treated tailings over time using different treatment methods. An important observation from Figure 10 is that reducing the proportion of CKD led to a decrease in the strength of the treated soil. This decline can be attributed to the higher proportion of gypsum, as CKD has a stronger strengthening effect compared to recycled gypsum. Conversely, increasing the admixture percentage in the tailings improved strength. After 7 days, the treated mixtures achieved UCS values in the range of 10–45 kPa, increasing to approximately 15–50 kPa after 14 days. Although these values are relatively low in absolute terms, they represent a substantial improvement compared to untreated tailings, which initially exhibit negligible strength due to their slurry-like condition. Such strength levels are consistent with early-stage stabilization requirements in upstream tailings dam construction. The observed increase in strength with curing time suggests continuing cementitious and pozzolanic reactions within the treated matrix. In the CKD–gypsum system, the alkaline environment generated by CKD is expected to facilitate activation and precipitation processes that improve interparticle bonding. Nevertheless, the precise nature of the reaction products, such as ettringite or calcium-silicate-hydrate-type phases, was not directly verified in the present work and therefore should be regarded as a plausible interpretation rather than a confirmed mineralogical finding [40].

It is noteworthy that the recycled gypsum underwent sieving through a #20 U.S. sieve, indicating the presence of coarse particles resembling fine sand. In contrast, CKD was sieved through a #40 U.S. sieve, indicating finer particles. As a result, the surface area of the gypsum particles was smaller than that of CKD, resulting in lower water absorption efficiency. Consequently, the outer surfaces of gypsum particles absorb water and set, while their inner parts absorb little. This characteristic causes soil treated with gypsum to exhibit a higher water content (Wc%) than soil treated with CKD or with finer gypsum particles.

Furthermore, Figure 16 demonstrates that the strength of the treated soil increased with longer curing times, progressing from 7 days to 14 days. However, the percentage increase in strength was approximately 10%, suggesting that most of the improvement occurred within the first 7 days. This aspect is beneficial for tailings-dam applications, as it facilitates early strength development.

#### 4.2.3. Effect of Commercial Gypsum Proportion on UCS

To evaluate the impact of different gypsum sources on the outcomes, two types of gypsum samples were utilized. The findings revealed a decrease in unconfined compressive strength with an increasing recycled gypsum proportion in the mixture, attributed to the superior effectiveness of CKD. However, it was crucial to investigate whether recycled gypsum adversely affected the strength of the treated tailings. Therefore, powdered gypsum, commonly known as plaster, was used instead of recycled gypsum for tailing treatment. Following the same sample preparation and curing procedure as previously described for the 10% admixture combination, nine samples were cast and cured for 7 days before testing.

The results obtained from the tests on specimens containing plaster (designated with the letter P) are compared in Figure 16 with those obtained using recycled gypsum. As shown in Figure 16, samples treated with admixtures containing plaster exhibited a higher UCS as the proportion of plaster increased. For example, the UCS of the treated tailings increased by over 100% when the CKD:plaster ratio ranged from 1:1 to 1:4.

These findings clearly highlight the efficacy of gypsum in tailing treatments. The inferior performance of recycled gypsum in tailing treatments can be attributed to two main factors: the limited surface area of recycled gypsum, which affects the hydrophilicity of gypsum particles, and the lower calcium oxide (CaO) content of recycled gypsum compared to plaster, as indicated by the XRF analysis results presented in Table 2.

Understanding the stress–strain behavior of treated tailings is essential for accurately modeling their performance as construction materials for tailings dams. Figure 8 depicts the stress–strain curve of a specimen treated with a 10% admixture (10-2). Similar stress–strain behavior was observed in all other specimens. While all specimens exhibited some degree of strain softening during shearing, no distinct peak–post-peak behavior was observed.

It was noted that the gypsum content did not clearly affect the cracking strain, as all samples developed cracks at approximately the same strain level. However, the inclusion of cementitious materials altered the typical 45-degree cracking plane to multiple cracking planes.

Due to its effective performance in treating clayey tailings, the CKD:B admixture at a 1:1 ratio was selected for potential applications in treating silty tailings (tailing 1). Two different percentages were evaluated for treating tailing 1, and the treated materials underwent oedometer tests to assess consolidation behavior and direct shear tests to determine strength parameters.

### 4.3. Direct Shear Tests

**Figure 17:**
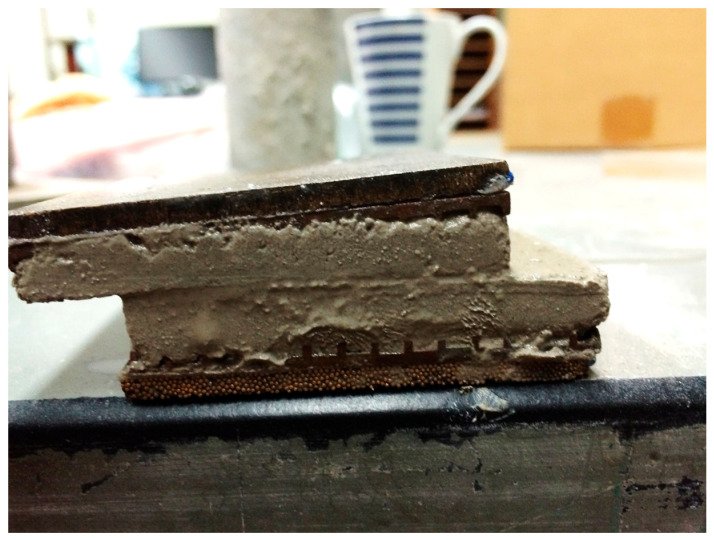
Direct shear sample after test completion.

**Figure 18:**
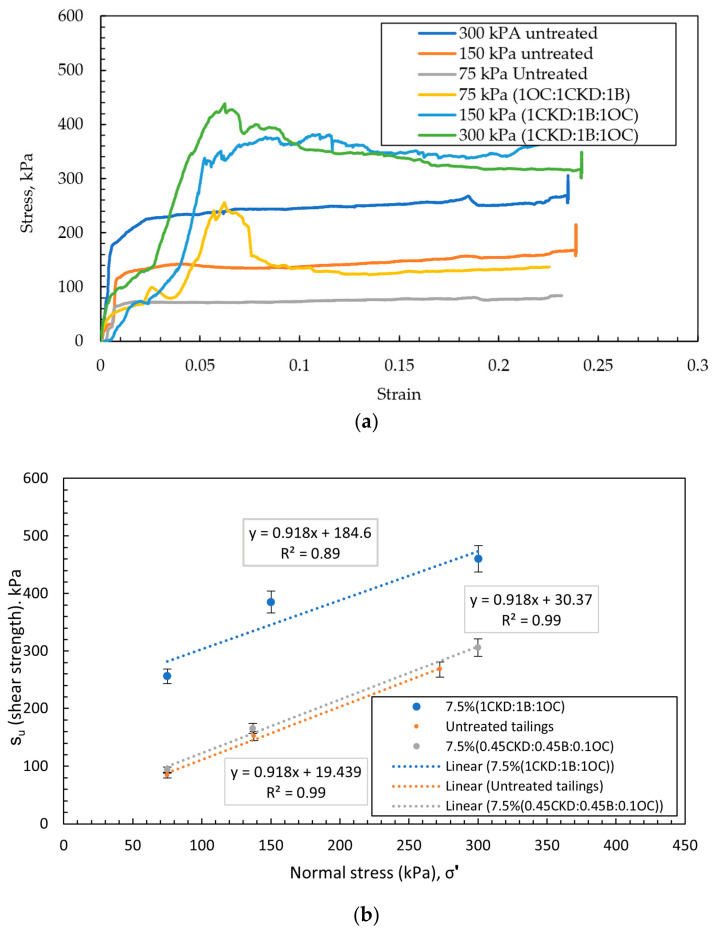
(**a**) Stress–strain relationship for untreated tailings and the 7.5% treated tailings; (**b**) DST on treated and untreated tailings.

Figure 17 shows the 7.5% (1CKD:1B:1OC) sample after shearing. Figure 18 presents the results of direct shear tests (DSTs) for both untreated and treated tailings. Each normal load increment was maintained for one hour to facilitate dissipation of excess pore pressure (EPP). While complete drainage in fine-grained tailings cannot be fully guaranteed, the selected loading duration and shear rate were adopted to approximate drained conditions in accordance with standard practice. Additionally, a slow strain rate of 0.03 mm/min was employed during shearing to minimize pore pressure buildup and ensure drained conditions. The figure compares the shear strength envelopes of untreated tailings and tailings treated with 7.5% admixtures containing varying ratios of CKD, B, and OC. As shown, the treated specimens exhibited significantly higher shear strength than the untreated samples, primarily due to the formation of cementitious bonds from the additives. The most effective blend—comprising equal parts CKD, B, and OC (1:1:1)—achieved a cohesion intercept of 184–218 kPa, based on a linear regression of the Mohr–Coulomb failure envelope. Figure 18a is the plot of the stress–strain curve of the untreated and treated tailings with a 7.5 commercial concentration of (1CKD:1B:1OC). The untreated tailings’ stress–strain curves depict the expected hyperbolic correlation: stiffness response in the initial stages of the test is succeeded by yielding and a level off at the limiting shear strength. The treated tailings, on the other hand, exhibit very low resistance at the start because of the nature of the preparation of the samples, which left cracks in the sample which were filled by the fine tailings to seal the cracks. On reaching the point where the stress was large enough to mobilize the improved sample, the stress–strain reaction at that point begins to pick up and exhibits significantly different trends because of cementation. The response exhibited is brittle in nature with a very steep stress–strain curve and associates with ultimate capacity which is the breaking of the cementation. This is accompanied by strain softening and residual strength is obtained at approximately 50–70% of the final resistance. Although in this circumstance the R^2^ was below 0.9, the shear tests were repeated to ensure the consistency of the measured strength parameters. The results obtained show that the failure envelope tends to become flatter at higher stress levels, indicating that the shear strength becomes progressively less dependent on effective stress. Such behavior is commonly observed in cemented geomaterials and stiff or hard clays, particularly under undrained loading conditions, where bonding and structural effects can reduce the sensitivity of shear strength to confining stress. The coefficient of determination obtained from the Mohr–Coulomb fitting (R^2^ = 0.8874) reflects a moderate level of scatter. Therefore, the Mohr–Coulomb failure envelope presented in this study should be interpreted as an engineering approximation of the shear strength behavior, while acknowledging the natural variability associated with the tested material. In contrast, the admixture combination of 0.45CKD:0.45B:0.1OC showed minimal improvement, increasing cohesion by only 11 kPa compared to the untreated material. This modest gain can be attributed to a higher dry density rather than chemical bonding, indicating limited pozzolanic or cementitious reactions in this ratio.

Although some variability was observed in the measured values due to the inherent heterogeneity of the tailing materials, the overall trends remained consistent across replicate specimens. The reported results represent the average behavior, and the relative differences between treatment conditions were significantly greater than the observed variability, supporting the reliability of the conclusions. A limitation of the present study is that environmental durability was not directly evaluated. In practice, modified tailings dams may be exposed to seasonal wet–dry cycling and, in cold regions, repeated freeze–thaw action. These processes may alter the bond integrity, microstructure, and hydraulic response over time. Accordingly, the present study should be interpreted as a comparative laboratory investigation of short-term stabilization behavior rather than a complete mechanistic or durability-based assessment. The selected treatment formulations were intended to identify practically promising mixtures for tailing improvement using industrial by-products and polymer-based additives. Further work is recommended to examine environmental durability under wet–dry and freeze–thaw cycling, optimize the role of OC in saturated conditions, and directly characterize the mineralogical products responsible for strength development.

The strength properties of treated and untreated tailings, as determined by shear strength tests, are provided in Table 6. These properties can be used to develop appropriate treatment schemes and numerical models to assess the stability of tailings dams treated with the proposed admixture combination.

## 5. Conclusions

This paper compared the performance of mine tailing stabilization by using standard additives, namely, cement kiln dust (CKD) and recycled gypsum, and a non-traditional EAP. Experiments at the laboratory, such as unconfined compressive strength (UCS), oedometer consolidation and direct shear experiments, were performed to determine the strength, stiffness, compressibility and hydraulic behavior enhancements of the treated tailings. This study provides a comparative evaluation of stabilization techniques rather than a statistically exhaustive dataset. Nevertheless, the observed trends were consistent across repeated tests, and the magnitude of improvement between treatments was significant. Future work will focus on expanding the experimental database and applying more rigorous statistical analyses. All in all, it can be concluded that the industrial by-products, namely CKD and gypsum, could be of great help in improving the geotechnical properties of mine tailings and provide an alternative long-term stabilization method.

Key findings:

- Blends of CKD–gypsum were found to substantially enhance the mechanical properties of tailings. These optimum mixtures promoted unconfined compressive strength (UCS), cohesion, and stiffness as compared to untreated tailings.
- The ideal level of treatment was about a 7.5 percent additive level.
- The compressibility of tailings was also significantly lowered after stabilization using CKD–gypsum blends. The compression index reduced significantly, which showed enhanced settlement and deformation resistance to applied loads.
- The values of UCS improved by over 100% when plaster was involved because of the greater content of calcium oxide and the smaller size of the particles.
- Despite the fact that the EAP enhanced the dry unit weight of the material, oedometer tests depicted enhanced compressibility due to the rubber-like structure of the polymer matrixes.
- The results of direct shear tests proved that the shear strength parameters significantly improved. There was also a significant improvement in cohesion in the treated samples which showed that there was stronger bonding between the particles as a result of cementitious reactions. The majority of the improvement in strength came about in the early curing period. Most of the UCS enhancement was noted in the first seven days which is beneficial in the practice construction of tailings dams and staged constructions of embankments.

Final remark: In general, the results indicate that CKD–gypsum stabilization is cost-effective, sustainable, and technically feasible for enhancing tailing stability, especially in upstream tailings dams.

## Figures and Tables

**Table 1:** General physical properties of tailings.

Index Parameters	Value
USCS of the tailings	ML
Mean particle size, D_50_ (mm)	0.045
Effective particle size, D_10_ (mm)	0.0175
Coefficient of uniformity, C_u_	3.4
Coefficient of curvature, C_c_	0.8
Liquid limit, LL (%)	48
Plastic limit, PL (%)	NP
Liquidity index, LI	1.34
Specific gravity, G_s_	2.69

**Table 2:** XRF analysis results.

	Recycled Gypsum		Plaster		Tailing 1		Tailing 2	
	Wt%	StdErr	Wt%	StdErr	Wt%	StdErr	Wt%	StdErr
CaO	53.7	0.25	67.48	0.23	44.05	0.25	13.72	0.17
SO_3_	44.91	0.25	26.7	0.22	1.72	0.07	1.34	0.06
SiO_2_	0.543	0.027	1.82	0.07	32.38	0.23	44.63	0.25
Fe_2_O_3_	0.268	0.013	0.133	0.007	1.88	0.07	18.13	0.19
Al_2_O_3_	0.192	0.019	0.591	0.029	10.81	0.16	10.82	0.16
MgO	0.168	0.008	2.48	0.08	4.2	0.1	3.48	0.09
K_2_O	0.0576	0.0029	0.371	0.018	2.36	0.08	5.01	0.11
TiO_2_	0.0463	0.0041	0.0482	0.0046	0.822	0.041	0.58	0.029
Sc	0.0287	0.0027	0.034	0.003	0.0201	0.0024	0	0
La	0.0243	0.0058	0.0344	0.0068	0.0187	0.0058	0.04	0.0049
Sr	0.0228	0.0012	0	0	0.068	0.0034	0	0

**Table 3:** Physical properties of the EAP used.

Properties	Resin	Part B	Accelerator	Hardener
Appearance	Clear liquid	White liquid	Clear liquid	White solid
Viscosity	5 mPa·s	12 mPa·s	2 mPa·s	--
Density	1.05 kg/L	1.01 kg/L	0.93 kg/L	Approx. 2.6 kg/L
Notes	--	--	at 20 °C	--

**Table 4:** Physical properties of the final EAP product.

Index Parameters	Value
Physical state	Liquid
Color	Transparent
Solvability in water	Solution
Viscosity (mPa.s)	13 (very low)
Flashing point	600 °C
Density (kg/L)	1.07 (20°)
Gel time (s)	0:48–8:46 depending on the accelerator
pH	Not measured

**Table 5:** Laboratory test matrix.

Category	% of Admi./Ms	Compound	Number of Samples			Purpose/Notes
			UCS	Oedometer	DST	
Untreated Tailings	0	T1	0	3	6	Define shear strength parameter
A. Emulsified acrylic polymer (EAP)	0.5	EAP 0.5%	0	3	0	Define optimal treatment percentage
	1	EAP 1%	0	3	0	
	2	EAP 2%	0	3	0	
B. Optimization study	5	1CKD:1B	6	-	-	Define optimal treatment percentage
	10	1CKD:1B, 1CKD:2B, 1CKD:4B	18	-	-	
	20	20-1	6	-	-	
C. Stiffness and strength evaluation	10	1CKD:1B	6	2	6	Little improvement when soaked in water
	7.5	0.45CKD:0.45B:0.1OC	-	2	6	Overcome the solubility of anhydrite, define shear strength parameters
		1CKD:1B:1OC	-	2	6	

**Table 6:** Tailings properties.

Material	c’	Φ’	Eoed.	k (m/s)
Untreated tailings	0	38	9400	9.12 × 10^−6^
Treated tailings (1:1:1)	184	38	300,284	6.01 × 10^−7^

## Data Availability

The data will be made available upon request.

## Glossary

s_u_	Undrained shear strength of the soil
MTs	Mine tailings
TSF	Tailings storage facility
EAP	Emulsified acrylic polymer
CKD	Cement kiln dust
OC	Ordinary Portland cement
B	Bassinate
c’	Soil cohesion
Φ’	Soil friction angle
e	Void ratio
ε_v_	Volumetric strain
ε_s_	Shear strain
DST	Direct shear test
UCS	Unconfined compressive strength
XRF	X-ray fluorescence spectroscopy
ICP	Inductively coupled plasma
USCS	Unified soil classification system
σn’	Effective stress, *n* = 1, 2 and 3
Cc	Compression index
Cv	Coefficient of consolidation m^2^/s
k	Hydraulic conductivity (m/s)
Wc (%)	Water content
E_oed_	One-dimensional constrained modulus from oedometer testing
ESA	Effective stress analysis
EPP	Excess pore water pressure

## References

[1] Alsharedah Y., Naggar M.H.E. Effects of tailings treatments scheme on tailings dam. Proceedings of the 9th ICEG.

[2] Radchuk O., Stefanyshyn D. (2025). Spatial Variability and Statistical Analysis of Tailings Dam Soil Properties. IOP Conf. Ser. Earth Environ. Sci..

[3] Alsharedah Y.A. (2015). Slope Stability Enhancement of an Upstream Tailings Dam: Laboratory Testing and Numerical Modelling. Master’s Thesis.

[4] Chen Y., Wei Z. (2023). Stability of Tailings Dam Constructed by both Upstream and Centerline Methods. Geotech. Geol. Eng..

[5] Rico M., Benito G., Díez-Herrero A. (2008). Floods from tailings dam failures. J. Hazard. Mater..

[6] Davis M., Martin T., Lighthall P. (2002). Mine Tailings Dams: When Things Go Wrong. Proceedings of the ASDSO Conference Papers, Las Vegas, NV, USA, 28–30 March 2000.

[7] Yin G., Li G., Wei Z., Wan L., Shui G., Jing X. (2011). Stability analysis of a copper tailings dam via laboratory model tests: A Chinese case study. Miner. Eng..

[8] Alsharedah Y.A., El Naggar M.H., Ahmed A. (2023). Improving Tailings Dam Safety via Soil Treatment. Sustainability.

[9] Armstrong M., Langrené N., Petter R., Chen W., Otávio P.C. (2019). Accounting for tailings dam failures in the valuation of mining projects. Resour. Policy.

[10] Arun L., Sujatha E.R., Baldovino J.A., Nuñez de la Rosa Y.E. (2024). Microcrystalline Cellulose—A Green Alternative to Conventional Soil Stabilizers. Polymers.

[11] Vick S.G. (1990). Planning, Design, and Analysis of Tailings Dams.

[12] Bjelkevik A. (2005). Water Cover Closure Design for Tailings Dams. Master’s Thesis.

[13] Liu L., Huang H. (2012). Application of water-soluble polymers in soil quality improvement. Civ. Eng. Urban Plan..

[14] Guedes J.P.C., Silvani C., de Azambuja Carvalho J.V., Wagner A.C., de Sousa Silva J.P., Consoli N.C. (2024). Mechanical Behaviour of Fibre-Reinforced Cemented Iron Ore Tailings Across the Compaction Curve. Geotech. Geol. Eng..

[15] Vlček J., Gago F., Mihálik J., Malík F., Bahleda F., Prokop J. (2024). Investigation of dynamic effect of rapid impact compaction. Sci. Rep..

[16] Cai J., He Z., Xu B., Yu M. (2023). The effect of PVD layout on the consolidation characteristics of dredged slurry under vacuum preloading. Front. Mar. Sci..

[17] Fan L., Xun Z., Peng S. (2023). A Comparative Case Study on Drainage Consolidation Improvement of Soft Soil under Vacuum Preloading and Surcharge Preloading. Appl. Sci..

[18] Ren Q., Wang Y. (2024). Theoretical and experimental study on the consolidation of soil with continuous drainage boundary under electroosmosis–surcharge preloading. Sci. Rep..

[19] Jiang L., Qin A., Gong J. (2023). Analytical models for consolidation of unsaturated composite foundation improved by impermeable columns and PVDs. Structures.

[20] Sun S.-W., Zhu B.-Z., Wang J.-C. (2013). Design method for stabilization of earth slopes with micropiles. Soils Found..

[21] Xue Z., Zhang W., Zhao X., Meng F., Qin F., Xiao G., Nie Z., Chen J. (2024). Utilization of cement deep mixing pile for soft soil foundation: A malaysian case study. Front. Mater..

[22] Abankwa B., Razavi M., Otoo R., Armah A., Donkor S. (2025). Effects of Sand–Cement Columns on Primary Consolidation Settlement. Appl. Sci..

[23] Rochanavibhata U., Noorak A., Jongpradist P., Makaratat N., Jing G., Jamsawang P. (2024). Consolidation Behavior of Silty Sand Improved by Stone Column. Transp. Infrastruct. Geotechnol..

[24] Kamei T., Ahmed A., El Naggar M.H. (2017). Performance of Ground Improvement Projects Incorporating Sustainable Reuse of Geo-composite Wastes. Transp. Geotech..

[25] Simon D., Murray W., Grabinsky M.W., Bawden W. Effect of polycarboxylated acrylic acid polymer-based superplasticizer on cemented paste backfill. Proceedings of the 14th Pan-American Conference on Soil Mechanics and Geotechnical Engineering & 64th Canadian Geotechnical Conference.

[26] Sujatha E.R., Pratheeba V.R., Baldovino J.D.J.A., Nunez de la Rosa Y.E. (2025). Application of Gelatin for Sustainable Stabilization of Low-Compressible Silt–Clay Mixtures: Geotechnical Behavior and Carbon Emission Considerations. Polymers.

[27] Ateş A. (2013). The effect of polymer-cement stabilization on the unconfined compressive strength of liquefiable soils. Int. J. Polym. Sci..

[28] Adajar M.A., Tan J., Adriano A., Vera S.B.D., Manabat J.V., Navarro H. (2025). Agar Biopolymer as a Sustainable Alternative Binder to Enhance the Strength of Low-Plasticity Soil. Polymers.

[29] Wang R., Ong D.E.L., Sadighi H., Goli M., Xia P., Fatehi H., Yao T. (2025). Optimizing Soil Stabilization with Chitosan: Investigating Acid Concentration, Temperature, and Long-Term Strength. Polymers.

[30] Baldovino J.d.J.A., de la Rosa Y.E.N., Calabokis O.P., Vergara J.A.A., López L.C.S. (2025). Geotechnical Behavior of Xanthan Gum-Stabilized Clay Reinforced with Polypropylene Fibers. Polymers.

[31] Zhang J., Liu J. (2023). A Review on Soils Treated with Biopolymers Based on Unsaturated Soil Theory. Polymers.

[32] Ahmed A., Nagy N.M., El Naggar M.H., Kamei T. (2018). Stabilisation of soft soil with recycled plaster admixtures. Ground Improv..

[33] Bertero A., Leoni F.M., Filz G., Nozu M., Druss D. (2012). Bench-Scale Testing and QC/QA Testing for Deep Mixing at Levee LPV 111. Grouting and Deep Mixing 2012.

[34] (2011). Standard Practice for Classification of Soils for Engineering Purposes (Unified Soil Classification System).

[35] (2017). Standard Test Methods for Liquid Limit, Plastic Limit, and Plasticity Index of Soils.

[36] (2011). Standard Test Methods for One-Dimensional Consolidation Properties of Soils Using Incremental Loading.

[37] Zinchenko A., Sakai T., Morikawa K., Nakano M. (2022). Efficient stabilization of soil, sand, and clay by a polymer network of biomass-derived chitosan and carboxymethyl cellulose. J. Environ. Chem. Eng..

[38] (2017). Standard Test Method for Direct Shear Test of Soils Under Consolidated Drained Conditions.

[39] Umar I.H., Lin H., Ibrahim A.S. (2023). Laboratory Testing and Analysis of Clay Soil Stabilization Using Waste Marble Powder. Appl. Sci..

[40] Yin G., Zhang Q., Wei Z., Jiang C., Wang M., Geng W. (2011). Development and application of observation testing apparatus for micromechanics and deformation of tailings. Yanshilixue Yu Gongcheng Xuebao/Chin. J. Rock Mech. Eng..

[41] Benson C.H., Kucukkirca I.E., Scalia J. (2010). Properties of geosynthetics exhumed from a final cover at a solid waste landfill. Geotext. Geomembr..

[42] Hu L., Wu H., Zhang L., Zhang P., Wen Q. (2017). Geotechnical Properties of Mine Tailings. J. Mater. Civ. Eng..

[43] Wu J., Min Y., Li B., Zheng X. (2021). Stiffness and Strength Development of the Soft Clay Stabilized by the One-Part Geopolymer under One-Dimensional Compressive Loading. Soils Found..

[44] Karim H., Al-Soudany K. (2018). Improving Geotechnical Properties of Clayey Soil Using Polymer Material. MATEC Web Conf..

[45] Consoli N.C., Foppa D., Festugato L., Heineck K.S. (2007). Key Parameters for Strength Control of Artificially Cemented Soils. J. Geotech. Geoenviron. Eng..

[46] Bouazza A. (2002). Geosynthetic clay liners. Geotext. Geomembr..

[47] Reid D., Fourie A. (2016). Laboratory Assessment of the Effects of Polymer Treatment on Geotechnical Properties of Low-Plasticity Soil Slurry. Can. Geotech. J..

[48] Azzam W.R. (2014). Behavior of modified clay microstructure using polymer nanocomposites technique. Alex. Eng. J..

